# Life-saving left completion pneumonectomy for lung abscess due to *Pseudomonas aeruginosa*: a case report

**DOI:** 10.1186/s44215-026-00242-9

**Published:** 2026-02-07

**Authors:** Shorin Matsumoto, Noriko Hiyama, Kento Fukumoto, Tomoki Tamura, Hiroshi Goto, Jun Matsumoto

**Affiliations:** 1 Department of Thoracic Surgery, NTT Medical Center Tokyo, 5-9-22 Higashi- Gotanda, Shinagawa-ku, Tokyo, 141-8625 Japan; 2 Department of Cardiovascular Surgery, NTT Medical Center Tokyo, 5-9-22 Higashi- Gotanda, Shinagawa-ku, Tokyo, 141-8625 Japan

**Keywords:** Pseudomonas aeruginosa, Lung abscess, Community-acquired pneumonia, Lobectomy, Completion pneumonectomy

## Abstract

**Background:**

*Pseudomonas aeruginosa* is typically an opportunistic pathogen but, on rare occasions, may cause community-acquired pneumonia in healthy adults, often resulting in rapid and severe clinical deterioration. This case report details the successful management of a *P. aeruginosa*-induced *community-acquired lung abscess in a patient who had fully returned to normal daily life 1 year and 5 months after a left upper lobectomy.*

**Case presentation:**

A 63-year-old woman underwent video-assisted thoracoscopic left upper lobectomy with lymph node dissection for lung squamous cell carcinoma 1 year and 5 months prior. She received four courses of adjuvant chemotherapy postoperatively and remained recurrence-free. The patient presented with cough and fever and was diagnosed with a pulmonary abscess characterized by extensive infiltrative shadowing with cavitation in the left lower lobe. She developed septic shock requiring vasopressor support. On hospital day 3, worsening pulmonary infiltrates and altered consciousness prompted an emergency left completion pneumonectomy for infection source control, *as antibiotic therapy alone was insufficient.* Postoperative culture confirmed *Pseudomonas aeruginosa*. Although two subsequent hematoma evacuations and drainage were required, the patient recovered and was discharged on postoperative day 39.

**Conclusion:**

We present a rare case of *P. aeruginosa* community-acquired lung abscess complicated by septic shock. *In rapidly progressive cases*,* early consideration of P. aeruginosa infection*,* prompt administration of antibiotics with anti-P. aeruginosa activity*,* and early assessment for surgical intervention may improve patient outcomes.*

## Background

*Pseudomonas aeruginosa* is a well-known pathogen responsible for opportunistic infections. Although rare, several case reports have documented *P. aeruginosa*-induced community-acquired pneumonia (CAP) in previously healthy individuals, often with rapid and fatal progression [1–3]. However, there are few reports of *P. aeruginosa*-caused community-acquired lung abscess (CALA) in healthy adults. We present a case of *P. aeruginosa* CALA that progressed rapidly to septic shock, despite *the patient being in good general condition*, occurring 1 year and 5 months after left upper lobectomy for lung cancer.

## Case presentation

A 63-year-old woman underwent video-assisted thoracoscopic left upper lobectomy with lymph node dissection (ND2a-1) for left upper lobe pulmonary squamous cell carcinoma (pT3 [intrapulmonary metastasis] N1M0-IIIA). She completed four cycles of adjuvant chemotherapy with cisplatin and vinorelbine, which ended 1 year and 1 month prior to the current presentation, and remained recurrence-free. She had no medical history other than lung cancer, took no regular medications, and had no history of smoking or alcohol consumption. Her serum albumin level was 3.9 g/dL, indicating that her nutritional status was adequate, and her white blood cell count was within the normal range. Based on these findings, she was considered not to be in an immunosuppressed condition.

Four days before admission, she developed cough and persistent fever (38–40 °C). She was hospitalized due to poor oral intake. Upon examination, she was in a pre-shock state with blood pressure of 60/40 mmHg, a heart rate of 106 beats per minute, and a fever of 38.0 °C. Oxygen saturation was 99% on room air. Coarse crackles were noted in the left lung field.

**Fig. 1:**
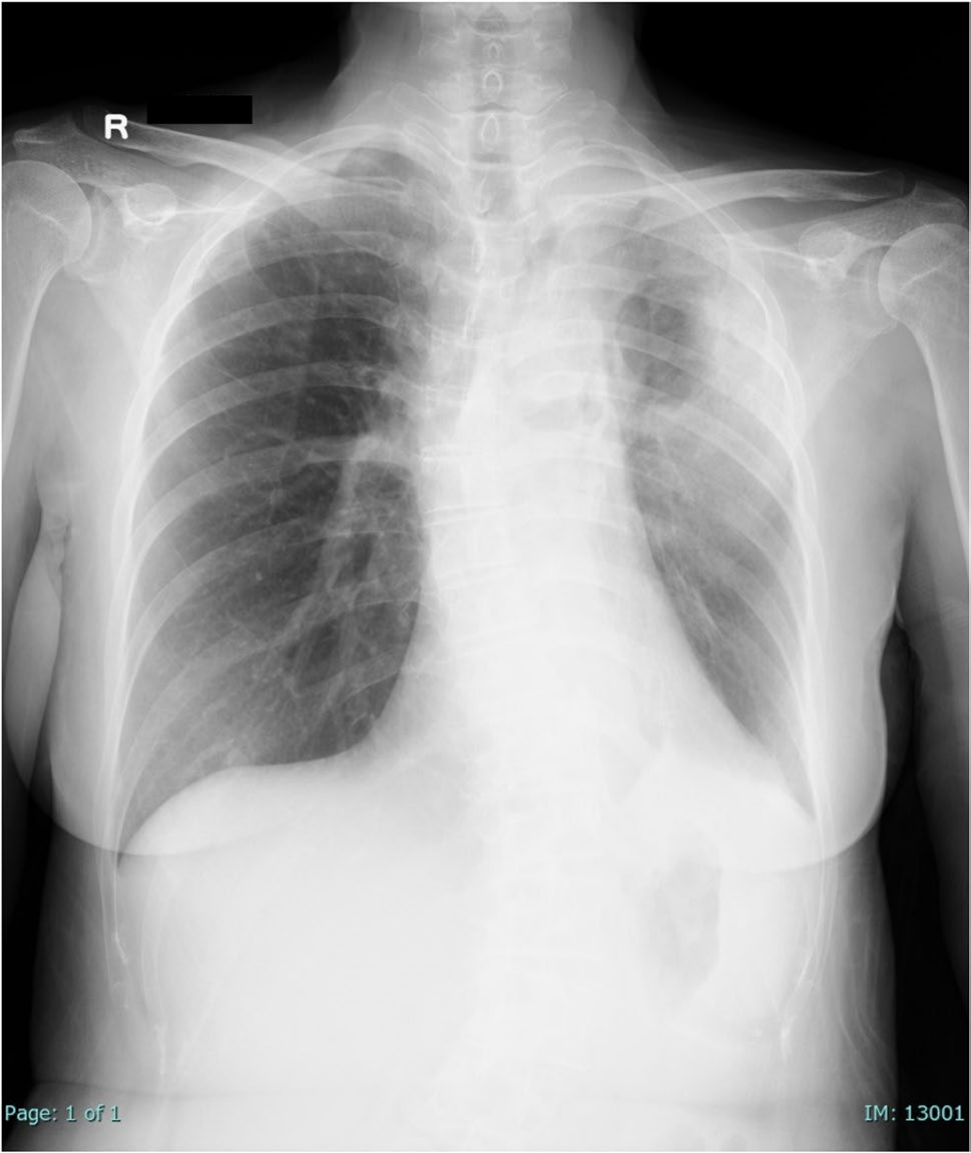
Chest X-ray image at admission, clearly showing infiltrative shadowing in the left lung

**Fig. 2:**
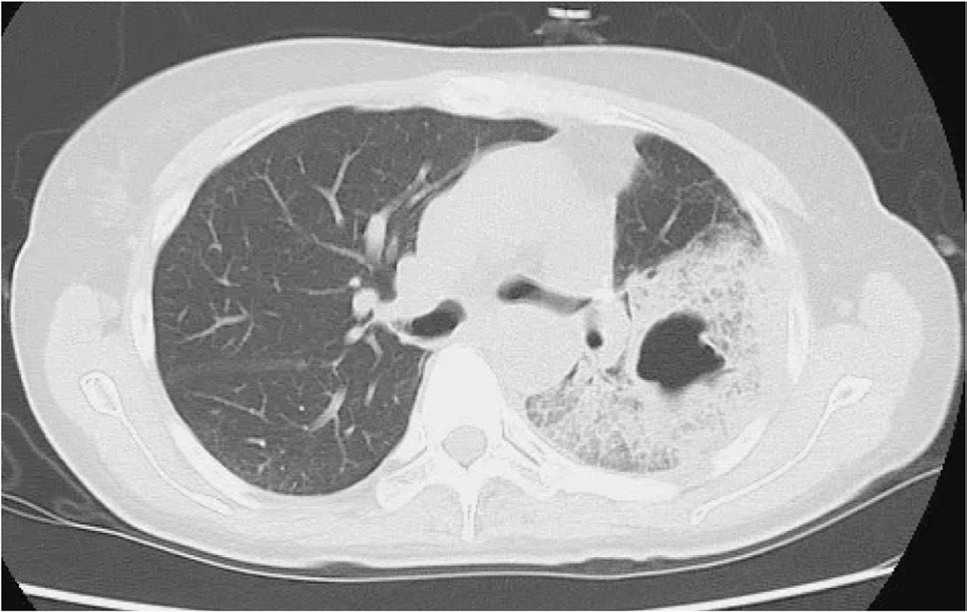
Chest computed tomography shows cavitation in the left lower lobe with surrounding infiltrates

Laboratory results showed a white blood cell count of 17,000/µL, hemoglobin of 9.9 g/dL, platelet count of 249,000/µL, and a C-reactive protein level of 33.78 mg/dL. Mild renal dysfunction, likely due to dehydration, was also noted. Sputum and blood cultures were negative. Chest X-ray taken during a routine visit four days prior to admission showed no abnormal lung shadows. However, chest X-ray upon admission showed clear infiltrative shadowing in the left lung (Fig. 1). Chest computed tomography (CT) demonstrated a cavitary lesion in the left lower lobe with surrounding infiltrates (Fig. 2).

**Fig. 3:**
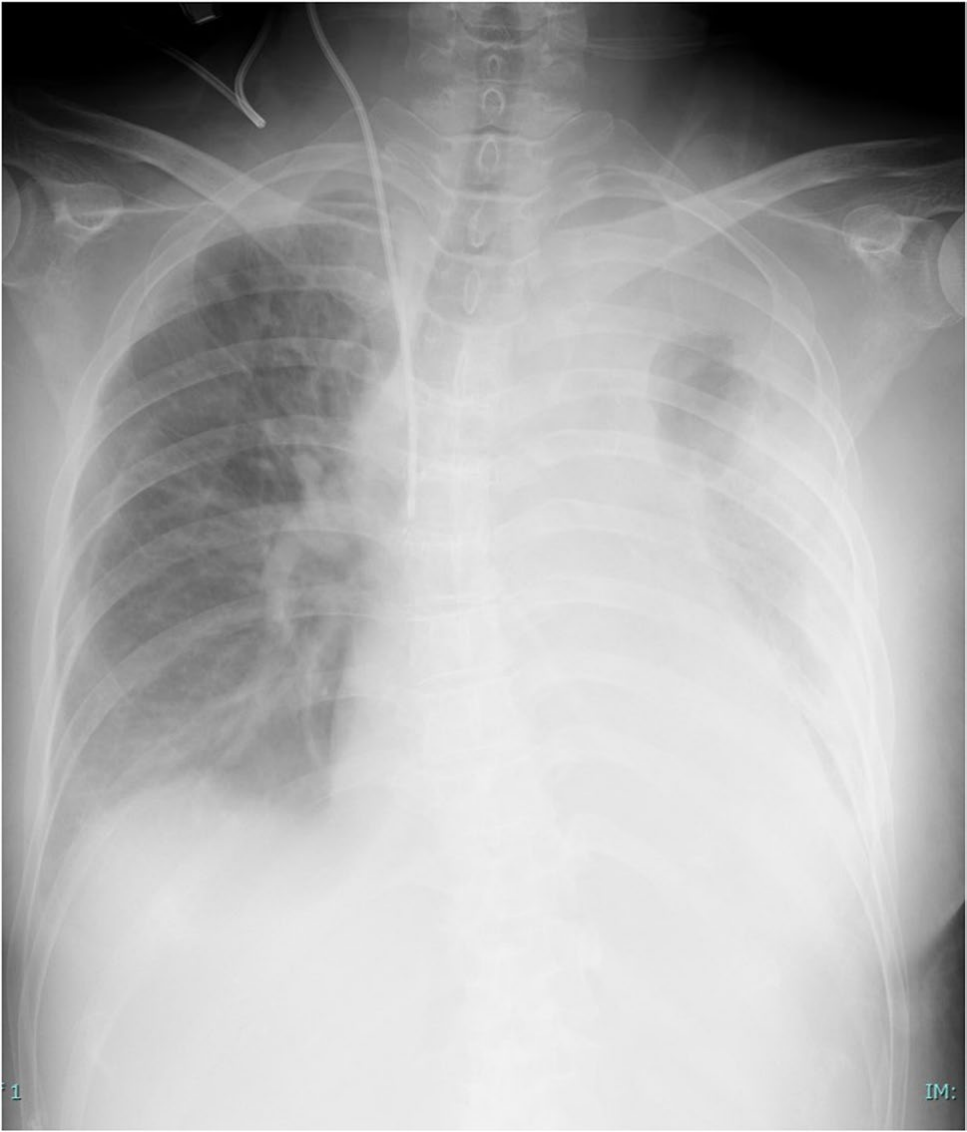
Chest X-ray on hospital day 3 shows marked exacerbation of left lung infiltration

The patient was diagnosed with lung abscess accompanied by pneumonia caused by abscess contents. Empiric antibiotic therapy with ampicillin/sulbactam and vasopressor support with noradrenaline (0.04 µg/kg/min) were initiated. Despite gradual escalation of noradrenaline up to 0.19 µg/kg/min by noon on hospital day 2, her blood pressure remained poorly responsive at 79/53 mmHg; therefore, vasopressin was added at a dose of 0.1 mU/min. Although the patient became progressively less active, no overt disturbance of consciousness was evident by the end of hospital day 2. In the early morning of hospital day 3, her blood pressure showed only minimal improvement to 97/68 mmHg and remained unstable, while oxygen demand increased to 2 L/min. Significant exacerbation of left lung infiltration on X-ray were noted (Fig. 3). In addition, the white blood cell count had worsened from 17,000/µL on admission to 23,900/µL. Based on these findings, antibiotic efficacy was judged to be insufficient, and antimicrobial therapy was escalated to a combination of meropenem and vancomycin. However, the patient subsequently developed worsening disturbance of consciousness: her Glasgow Coma Scale score, which had been E4V5M6 in the early morning, declined to E3V4M5 by later that morning, representing a decrease of more than two points within a short period. Given this rapid neurological deterioration and the inability to achieve adequate infection control with medical therapy alone, the situation was considered emergent. Therefore, urgent removal of the infectious focus was deemed necessary, and the decision was made to perform emergency left completion pneumonectomy.

An antero-lateral thoracotomy was performed, revealing moderate bloody pleural effusion. The left lower lobe showed empyema and inflammatory changes, making adequate collapse difficult despite selective lung ventilation. Extensive adhesions were present, especially at the site of the pulmonary artery, left main bronchus, and distal descending thoracic aorta, requiring meticulous dissection. Pus was observed at the site of the lung abscess and sent for culture. Because intraoperative hypotension and dense adhesions made the aortic wall difficult to identify, an unexpected aortic wall injury occurred, requiring cardiopulmonary bypass and reconstruction of the distal aortic arch with an artificial vessel graft. Following successful left completion pneumonectomy, the site of the graft and the bronchial stump were covered with omentum. The procedure lasted 10 h and 29 min, with blood loss of 2478 mL. Culture of the lung abscess confirmed *P. aeruginosa* as the pathogen. Histopathology showed extensive abscess formation with pulmonary necrosis. A 10-mm recurrent squamous cell carcinoma was identified in the pulmonary arterial wall without bronchial invasion.

After surgery, the patient required intensive care. Improvement in hypotension was noted the following day, and vasopressors were discontinued by the second day. Although the patient’s consciousness and sepsis improved, postoperative hemothorax required two thoracotomies. Her condition steadily improved, she began oral levofloxacin (LVFX) on day 39, and she was discharged uneventfully soon after. Oral LVFX was discontinued 1 month after discharge, without recurrence. She later underwent homograft vessel replacement and esophageal fistula closure for suspected infection and remains alive 18 months after the last operation.

## Discussion and conclusions

*P. aeruginosa* rarely causes CAP in healthy individuals without predisposing conditions such as immunodeficiency, neutropenia, bronchiectasis, chronic obstructive pulmonary disease, or recent hospitalization [1–4]. Despite undergoing lung cancer surgery and postoperative chemotherapy, the patient had been in good health for more than a year without recurrence or other risk factors; therefore, *P. aeruginosa* was not initially suspected. While *P. aeruginosa* accounts for 0.1%–5.2% of pathogens in hospitalized CAP, *P. aeruginosa*-induced CAP is even lower in healthy individuals [5–7]. However, *P. aeruginosa* CAP in healthy individuals is known to follow a rapidly progressive clinical course, with mortality reported at 17.6% overall and up to 33% in healthy adults [3, 7–9].

*P. aeruginosa* is known for its ability to acquire drug resistance, although community-acquired strains generally show greater susceptibility to ciprofloxacin (CPFX) and ceftazidime than nosocomial strains [2]. In the present case, the strain was susceptible to multiple antibiotics. Nevertheless, in cases of severe pneumonia requiring ICU admission, regardless of underlying conditions, guidelines recommend initial combination therapy with a broad-spectrum β-lactam and a fluoroquinolone such as CPFX or LVFX [2, 10, 11]. Early transition to this regimen has been associated with improved survival in life-threatening *P. aeruginosa* CAP [2, 3, 12].

Cases of *P. aeruginosa* CALA in healthy individuals are extremely rare [1, 13]. *P. aeruginosa* community-acquired pneumonia in otherwise healthy adults is known to be prone to severe disease, and *P. aeruginosa* is inherently difficult to treat with antimicrobial therapy. In the present case, although the patient had previously undergone a left upper lobectomy and the bronchus of the left lower lobe was not completely obstructed, bronchial kinking may have been present, which could not be ruled out as a contributing factor to the exacerbation of the lung abscess and pneumonia. On the other hand, although recurrence of lung cancer was identified in the resected specimen, the recurrent lesion was small, measuring 10 mm in diameter, and was therefore considered unlikely to have contributed to immunodeficiency or to the rapid clinical deterioration observed in this case.

Surgical intervention for lung abscesses is considered appropriate in cases when antibiotic therapy fails, when complications such as hemoptysis, empyema, or bronchopleural fistula develop, when underlying lung cancer is suspected, or when conservative therapy for more than 8 weeks fails to resolve abscesses larger than 6 cm [6, 14]. In the present case, despite the strain being susceptible to multiple antibiotics, infection control could not be achieved with antibiotic therapy alone. Additional drainage was considered; however, the abscess had largely drained spontaneously into surrounding lung parenchyma, leaving a cavitary lesion, and no empyema was evident. Thus, neither transthoracic nor transbronchial drainage was expected to be effective. Given the rapid deterioration, the lack of feasible alternative treatment options, the absence of evident tumor recurrence, and the expectation that long-term prognosis could be improved through definitive infection control, we proceeded with completion pneumonectomy.

In conclusion, *P. aeruginosa* CALA can occur even in the absence of evident immunosuppression and may follow a fulminant clinical course. In rapidly progressive cases, early consideration of *P. aeruginosa* infection, prompt administration of antibiotics with anti-*P. aeruginosa* activity, and early assessment of drainage strategies, including surgical intervention, are crucial for improving prognosis.

## Data Availability

Data sharing is not applicable to this article as no datasets were generated or analysed during the current study.

## Abbreviations

CAP: Community-acquired pneumonia
CALA: Community-acquired lung abscess
CT: Computed tomography
LVFX: Levofloxacin
CPFX: Ciprofloxacin
